# Bacteriological profiles and drug susceptibility of Streptococcus isolated from conjunctival sac of healthy children

**DOI:** 10.1186/s12887-020-02203-9

**Published:** 2020-06-22

**Authors:** Ruili Ke, Min Zhang, Qin Zhou, Yunfei Yang, Ruifen Shen, Huipin Huang, Xiangrong Zhang

**Affiliations:** 1 Department of Ophthalmology, The People’s Hospital of Longhua, 38 Jianshe East Road, Shenzhen, 518000 China; 2 Research Institute of Shenzhen Children’s Hospital, Shenzhen, China

**Keywords:** Conjunctival sac; bacterial flora, Antimicrobial susceptibility, Antibiotics

## Abstract

**Background:**

To investigate bacterial flora and antibiotics susceptibility of *Streptococcus pneumoniae* isolated from the conjunctival sac of heathy children.

**Methods:**

Bacteria were isolated from the secretions of conjunctival sac of healthy children between 2015 and 2018. Antimicrobial susceptibility of isolated *S. pneumoniae* strains were determined using microbroth dilution method.

**Results:**

The sac secretions were collected from a total of 6440 children. 1409 samples presented bacterial growth, accounting for 21.8% of the samples. Among the 22 bacterial species isolated, 528 samples presented Gram-positive *Staphylococcus* spp. growth, accounting for 37.4% of the isolates, followed by *Corynebacterium* spp., counting for 30% of the isolates and *Streptococcus pneumoniae,* counting for 21.4% of the isolates. Antibiotics susceptibility tests showed that the majority of *S. pneumoniae* isolates were sensitive to most antibiotics tested. However, 72.8 and 81.2% of the isolates were resistant to erythromycin and tetracycline, respectively, and over 10% of them were resistant to gentamicin, tobramycin and rifampicin.

**Conclusions:**

The bacterial flora of healthy children is mainly consisted of Gram-positive bacteria belonging to *Corynebacterium* spp. and *Streptococcus* spp.; most of *S. pneumoniae* isolates were sensitive to antibiotics except erythromycin and tetracycline.

## Background

The conjunctival sac is the space bound between the palpebral and bulbar conjunctiva. It directly contacts the external environment and is connected to the skin. Due to the structure of conjunctival sac, it inevitably exposes to bacterial sources and may get infected. However, the lacrimal fluid in the conjunctival sac contains antibacterial agents such as immunoglobulins and components of the complement pathways such as lactoferrin, lysozymes and B-lysin, which also have antimicrobial activity [1, 2]. Therefore, bacteria in the sac would be able to cause eye infections [3]**.** However, eye infections such as keratitis, dacryocystitis and endophthalmitis may occur when the eyes are subjected to trauma, surgery or compromised immunity [2, 4]. A better understand of microbial flora is important to develop appropriate health and preventive measures, particularly for children. Although there are a number of studies reporting the presence of bacteria in the conjunctival sac, these works were conducted in patients suffering from various eye diseases, such as Stevens - Johnson syndrome and cataracts [5, 6]. These works show that the microbial flora may change as a result of surgery [7, 8], use of antibiotics [9] and even seasonal change. For instance, it was found that conjunctival bacteria in patients undergoing cataract surgery presented a seasonal prevalence pattern where different bacteria were identified in different seasons [10]. In addition, use of antibiotics is also found to change drug resistance in conjunctival bacteria as well as the microbial flora [11, 12]. These findings suggest that it is necessary to investigate the microbial flora in specific population to design rationale strategy for eye infection control.

Streptococcus is an important pathogen that causes panophthalmitis [13] and other diseases [14]. However, the distribution and drug resistance of Streptococcus in the conjunctival sac of children are rarely reported. In this study, we investigated the distribution of bacteria, especially Streptococcus, in 6440 healthy, pre-school children who visited our hospital located in southern China and analyzed the drug resistance of *S. pneumoniae* isolates. The findings would help develop effective and preventive measures to minimize ocular infections and diseases in child.

## Methods

### Subjects

Conjunctival sac secretions of the conjunctival sac were collected from Children visiting our hospital between May 2015 and June 2018. They were aged 0 to 6 years and visiting the hospital for eye examination, elective strabismus, refraction, cataract surgery with no clinical signs of ocular infection. They were excluded if the eye and physical examination showed ocular infections, erythema, edema or other infections-related symptoms. Children who used systemic antibiotics or local antibiotics or eye infection controls within a month were excluded. In addition, children with systemic or immunodeficiency diseases, lacrimal sac, lacrimal abnormalities and other congenital diseases were also excluded. The protocol of this study was approved by the ethics committee of The People’s Hospital of Longhua in accordance with the Declaration of Helsinki. Informed written consent was obtained from the parents of children for the study.

### Sampling collection and bacterial culture

The secretion samples were collected using sterile cotton swabs moistened with sterile saline after local cleaning with sterile saline. Secretion were collected from the inside to outside surfaces of the conjunctival fornix by gently pressing and kneading the swabs from one randomly selected eye. Care was taken not to touch the edge of the eyelid during the procedure and to ensure that the child does not blink. The collected specimens were immediately inoculated on blood and chocolate plates and incubated at 35 ± 2 °C for 24 h. For chocolate plates, culture was performed in 5% CO_2._ The cultures were smeared, stained and examined under microscope to identify the bacteria. Bacterial strains were identified using *Micro Scan* Autoscan-4 system (Siemens Healthcare, Germany).

### Antibiotic resistance assay

Antimicrobial susceptibility tests were performed according to the Clinical and Laboratory Standards Institute (CLSI) protocols [15]. The assays were conducted on an automatic *TDR*-200B *Bacterial and Antibiotics Susceptibility Analyzer (Jinyang Technology, Beijing, China). Broth microdilution method was used to assay the susceptibility of bacterial stains to antibiotics. Staphylococcus aureus* (ATCC29213), *S. pneumoniae* (ATCC49619), *H. influenza* (ATCC49247), *Pseudomonas aeruginosa* (ATCC27853), *Escherichia coli* (ATCC25922), *Enterococcus faecalis* (ATCC29212) were used as quality control strains. The susceptibility of tested strains were classified according to the CLSI standards [15].

### Statistical analysis

Data were analyzed using Microsoft Excel 2003. The normality of distribution of continuous variables was tested by one-sample Kolmogorov-Smirnov test. Continuous variables with normal distribution were presented as mean ± standard deviation (SD); Means of 2 continuous normally distributed variables were compared by independent samples Student’s t test. The frequencies of categorical variables were compared using Pearson χ^2^. A value of *P* < 0.05 was considered significant.

## Results

### Isolation rate

A total of 6440 samples were collected and analyzed from 3100 (48.1%) girls and 3340 (51.9%) boys, with an average age of 3.12 ± 1.44. A total of 1409 isolates were obtained with an overall isolation rate of 21.8%. Among them, 646 (45.8%) were from girls and 763 (54.2%) were from boys. The isolation rates were 18.3, 22.2 and 20.1% for children between ages 0 and 2, 2 and 4, and 4 and 6, respectively, and were statistically similar (*P* > 0.05) between the age groups.

### Bacterial isolates

Based on microbiological analysis with aid of *MicroScan* Autoscan-4 system, twenty-two bacterial species were identified. Among them, 528 isolates were Gram-positive *Staphylococcus spp*. (including *S. epidermidis, S. aureus, S. hominis* and *S. haemolyticus*) counting for 37.4% of all isolates*,* followed by *Corynebacterium spp*., counting for 30% of the isolates and *Streptococcus pneumoniae,* counting for 21.4% of the isolates (Table 1). In other Gram-positive bacteria, *Mycobacterium xerosis conjunctiva* was the most common one (*n* = 17). When the bacterial species in the Gram-positive samples were further categorized, it was found that *S. epidermidis* and *S. saprophyticus* accounted for 21.6 and 8.5% of the 1409 children isolates.

**Table 1:** Isolation of conjunctival bacteria from healthy children aged 0 to 6 years

Bacteria	No. isolates	Percentage
		(%)
Gram-positive		
Corynebacterium spp.	422	30.0
*Staphylococcus epidermidis*	305	21.6
Staphylococcus saprophyticus	120	8.5
*Staphylococcus aureus*	68	4.8
Staphylococcus hominis	21	1.5
Staphylococcus haemolyticus	14	1.0
*Streptococcus pneumoniae*	301	21.4
Other Gram-positive bacteria	37	2.6
Gram-negative		0.0
Moraxella spp.	97	6.9
Other Gram-negative bacteria	24	1.7

Isolation rate of Gram-negative bacteria was 8.6%, and among them most were bacteria belonging to Moraxella spp. and *Neisseria gonorrhoeae* (*n* = 8), *Haemophilus influenzae* (*n* = 12), and *E. coli* (*n* = 4) were also detected (Table 1).

### Antimicrobial susceptibility

The antimicrobial susceptibility of *S. pneumoniae* isolates was assayed against 17 antibiotics and the results showed that the majority of isolates could be classified as susceptible to most antibiotics tested based on the CLSI standards [15] (Table 2). All over 95% isolates tested were susceptible to ofloxacin, ceftriaxone, vancomycin, linezolid and levofloxacin. However, 72.8 and 81.2% of them were resistant to erythromycin and tetracycline, respectively. In addition, over 10% of them were resistant to gentamicin, tobramycin and rifampicin and these isolates also had relatively higher percentages with intermediate susceptibility (Table 2).

**Table 2:** Antibiotic susceptibility of *Streptococcus pneumoniae* isolated from healthy children aged 0 to 6 years

Antibiotics	Total no. isolates tested	Susceptible		Intermediate		Resistant	
		No.	%	No.	%	No.	%
Penicillin	261	235	90.0	19	7.3	7	2.7
Hydrobenzylcillin	210	188	89.5	15	7.3	7	3.2
Gentamicin	222	178	80.2	21	9.5	23	10.4
Tobramycin	310	239	77	26	8.5	45	14.5
Erythromycin	220	57	25.9	3	1.3	160	72.8
Rifampicin	215	163	75.6	26	12.3	26	12.1
Chloramphenicol	255	181	71.1	0	0	74	28.9
Gatifloxacin	240	192	80.0	29	12.1	19	7.9
Ciprofloxacin	226	177	78.3	30	13.1	19	8.6
Ofloxacin	199	195	97.8	2	1.2	2	1
Ceftriaxone	190	183	96.2	4	2	3	1.8
Cefepime	200	184	92.2	10	5	6	2.8
Vancomycin	218	218	100	0	0	0	0
Linezolid	259	257	99.1	2	0.9	0	0
Tetracycline	288	35	12.1	19	6.7	234	81.2
Levofloxacin	287	286	99.5	0	0	1	0.5
Gatifloxacin	311	281	90.5	21	6.6	9	2.9

## Discussion

Previous studies have demonstrated that *Staphylococcus* and *Streptococcus* are the main pathogens of bacterial conjunctivitis, with *S. aureus* and *S. viridans* identified as the most common species in neonatal bacterial conjunctivitis [16, 17]. However, bacterial flora in healthy children is rarely reported. A better understanding of bacterial flora and their susceptibility to antibiotics is important for prevention and treatment of bacterial conjunctivitis and rationale use of preventive antibiotics in eye surgery for sterilization of conjunctival sac. Our work shows that Gram-positive corynebacterium, *Staphylococcus* and *Streptococcus* were the dominant bacteria in the conjunctival sacs and Gram-negative bacteria were rare. Most of the *S. pneumoniae* isolates were susceptible to antibiotics tested except erythromycin and tetracycline to which most of the *S. pneumoniae* isolates were resistant.

*Streptococcus* is commonly found in the mouth, nasopharynx and eye of healthy subjects [18]. Under normal conditions, it is not pathogenic. However, after eye surgery, under compromised immunity, due to abuse of antibiotics or changed ocular microenvironment, conjunctival *Streptococcus* may result in infections, leading to conjunctivitis and even the breakage of corneal tissue [19]. In addition, bacteria in the conjunctival sac may cause keratitis, dacryocystitis and endophthalmitis in traumatic and surgical conditions [2, 4].

A total of 1409 bacterial isolates were obtained from the conjunctival sac samples with a total isolation rate of 21.9%. The predominant species were Gram positive *Corynebacterium* spp., *Staphylococcus* spp*.* and *S. pneumoniae*. This bacterial profile is very different from that of adult, where *Streptococcus* is less frequently found in the conjunctival sac [20], suggesting that *Streptococcus* is not only common in the nasopharynx of children, but also in the conjunctival sac, especially in infants under 6 years old. In addition, the difference in bacterial profiles between children and adults may be attributed to their difference in the immunity, tear composition, tear fluid hydrodynamics, exposing environment, antibiotics use, bacterial flora in the skin and upper respiratory tract [21, 22]. Previous studies showed that a number of factors are playing role in protecting eye from bacterial infections, including antimicrobial peptides [23], antimicrobial proteins [24, 25] and innate defense pathways [26]. In addition, the bacterial flora may also change chronologically. For example, the patterns of bacterial pathogens in neonatal bacterial conjunctivitis in Southern China has changed during the past 15 years. The number of cases involving Gram-positive bacteria exhibited a decreasing trend, whereas those with Gram-negative bacteria showed a growing trend in children with acute neonatal bacterial conjunctivitis [27].

Better understanding of antibiotics resistance is important for rationale use of antibiotics in preventive and therapeutic measures for child. Since *S. pneumoniae* is not only an important pathogen that causes ocular infections [28] but also is associated with a high degree of morbidity and mortality in many countries around the world and is considered the main cause of death of millions children in the transition countries [29]. We tested the susceptibility of the isolates against commonly available antibiotics and we observed that the majority of *Streptococcus* isolates obtained from the conjunctival sac are sensitive to the antibiotics tested, particularly to ofloxacin, ceftriaxone, vancomycin, linezolid and levofloxacin. However, 72.8 and 81.2% of them are resistant to erythromycin and tetracycline, respectively. The rates are higher than those reported previously for infants aged 0 to 1 year [30].

Resistance to erythromycin and tetracycline have been higher [31, 32], it demonstrates the widespread presence of transposons in pneumococcal populations, typified by Tn*1545*. Previous studies showed insertion over time of resistance determinants, such as *erm*(B) for erythromycin and *aphA3* for kanamycin, into primitive gram-positive conjugative transposons carrying *tet*(M) and the integrase gene *int-Tn*, typified by Tn*916* [33, 34]. Therefore, for pre-operative local sterilization for eye surgery and for prophylaxes, aminosaccharides and quinolones are recommended and erythromycin and tetracycline should be avoided.

## Conclusions

Our study shows that *Streptococcus* as well as other Gram-positive bacteria are commonly present in the eye of healthy children. Although most pneumococcal isolates are sensitive to antibiotics, there are strains that are resistant to erythromycin and tetracycline, as well as gentamicin, tobramycin and rifampicin. These results could be used in the selection of empirical therapy if it is not possible to perform susceptibility testing and for developing public health strategies for pre-school children.

## Data Availability

The datasets used during the current study are available from the corresponding author on reasonable request.

## Abbreviations

CLSI: Clinical and Laboratory Standards Institute
SD: Standard deviation
